# Application Strategy and Research Progress of Large-Scale Population Drug Intervention in Malaria Control

**DOI:** 10.3390/tropicalmed11050113

**Published:** 2026-04-25

**Authors:** Zichao Cao, Yunan Gu, Guoming Li, Changsheng Deng

**Affiliations:** 1 Artemisinin Research Center, Guangzhou University of Chinese Medicine, Guangzhou 510405, China; 20241110445@stu.gzucm.edu.cn (Z.C.);; 2 School of Public Health and Management, Guangzhou University of Chinese Medicine, Guangzhou 510006, China

**Keywords:** malaria, large-scale population drug intervention, review, antimalarial strategy

## Abstract

Malaria is one of the major global public health issues. An estimated 282 million malaria cases occurred worldwide in 2024, and the overall prevention and control progress has stagnated or even reversed in some regions. Mass drug administration (MDA), as a potential strategy to accelerate malaria elimination, has regained attention. This paper reviews the evidence base, controversial focuses, and application strategies of MDA in malaria prevention and control. It aims to promote its scientific application in the elimination phase. MDA plays an important role in malaria prevention and control. However, this strategy is accompanied by core limitations such as long-term drug resistance risks, insufficient implementation sustainability, and a high failure rate of regional adaptation. It also faces challenges from multiple common malaria species, as well as the newly discovered Plasmodium knowlesi. We therefore propose an “MDA+” collaborative strategy integrating vaccines, digital monitoring, and cross-border cooperation, so as to optimize resource allocation, achieve full coverage control over various malaria parasites, and advance the global malaria elimination process.

## 1. Introduction

Malaria, as one of the core challenges in global public health, exhibits significant heterogeneity in its epidemiological patterns across different regions [1]. According to the World Malaria Report 2025 and related data, an estimated 282 million malaria cases were reported in 80 malaria-endemic countries worldwide in 2024. The number of malaria cases continues to rise, with sub-Saharan Africa bearing over 90% of the global burden of malaria cases and deaths. Meanwhile, some countries in Southeast Asia and South America face the complex situation of coexisting drug-resistant Plasmodium and resistant Anopheles mosquitoes. Among them, Nigeria (27%), the Democratic Republic of the Congo (12%), Uganda (5%), and Mozambique (4%) together account for nearly half of the global cases. In most of their rural areas, the annual entomological inoculation rate (EIR) has been consistently over 100, and the high transmission intensity makes it difficult for conventional vector control measures to work alone [2,3]. The gradual promotion of novel prevention and control tools, along with the achievements in elimination in certain regions, highlights the potential for control. However, the interplay of multiple threats poses severe challenges to the global elimination goal [4]. M. Barber [5] first reported the use of MDA for malaria control as early as 1932. Drugs such as chloroquine, primaquine, sulfadiazine, artemisinin, and ivermectin have subsequently been employed as part of antimalarial regimens. With the widespread use of chloroquine, MDA was incorporated into global malaria eradication programs but was subsequently marginalized due to the global spread of chloroquine resistance and implementation complexities [6]. In the 21st century, MDA, led by artemisinin-based combination therapies (ACTs), experienced a resurgence due to its high efficacy, good tolerability, and gametocidal activity. However, the path to recovery is still accompanied by unresolved scientific controversies and practical dilemmas. Failed cases such as long-term drug resistance evolution, epidemic resurgence, and the collapse of community compliance repeatedly warn that it is not a universal quick-fix solution [7,8]. A cluster randomized controlled trial in southern Tanzania [9] showed that MDA combined with long-lasting insecticidal nets (LLINs) reduced malaria incidence by 75% in high-transmission areas and significantly decreased subclinical infection loads. Maya Fraser et al. [10] used interrupted time series methods to analyze malaria incidence data in Zambia, evaluating the significant reduction in incidence rates of MDA in malaria-endemic and low-transmission areas. In Cambodia’s malaria elimination demonstration zone, MDA combined with active case detection successfully interrupted local transmission of Plasmodium falciparum (Pf) [11]. A meta-analysis demonstrated that MDA significantly impacts the incidence and prevalence of Plasmodium falciparum and Plasmodium vivax (Pv) infections [12], highlighting its critical role in malaria-endemic regions. However, failure cases are not uncommon. For instance, in an MDA implementation in a West African country, inadequate integration of local tribal cultural structures in community mobilization led to rumor dissemination and strong community resistance, resulting in actual medication coverage being below 30%―far below the 80% threshold required to interrupt transmission and failed to achieve the desired outcomes [13]. The root cause lies in the project team’s excessive reliance on the technical efficacy of medications while neglecting the social effectiveness of public health interventions, coupled with a lack of long-term community engagement and two-way communication mechanisms. The solution requires integrating traditional community leaders into the core decision-making and implementation processes. Additionally, logistical disruptions caused by weak health systems often prevent the timely delivery of medications to remote villages [14]. The core issue stems from the severe misalignment between the high demands of quasi-military operations like MDA and the low-level capabilities of routine healthcare systems. The solution involves leveraging technological and model innovations to overcome physical bottlenecks, thereby fundamentally advancing the integration and strengthening of supply chains. Meanwhile, the ACTs used by MDA degrade under tropical conditions, leading to inefficacy and reduced malaria resistance [15]. The root cause lies in short-sightedness regarding environmental constraints and the lack of fundamental predictions and response strategies for drug stability under tropical extreme climates. Improvements include the mandatory adoption of heat-resistant formulations with high stability specifically designed for tropical conditions, as well as the strategic pre-positioning of medications at regional centers prior to the rainy season. This study systematically reviews the historical context of MDA, focusing on regional disparities, lessons learned from failures, and cost-effectiveness in tropical areas. It evaluates safety ethics and drug resistance risks, explores its long-term uncertainties, sustainability deficiencies, and practical limitations, and ultimately proposes an “MDA+” integrated strategy framework for the elimination phase.

## 2. Literature Search Strategy

The literature was retrieved from PubMed, MEDLINE, Web of Science, and Sci-hub using the English keywords: malaria, severe malaria, mass drug administration, MDA, fMDA, tMDA, Plasmodium falciparum, Plasmodium vivax, drug resistance, cost-effectiveness, ethics, and implementation. The search was conducted using a combination of subject headings and free-text terms, with no restrictions on language or study design, covering the period from database inception to 1 September 2025. The inclusion criteria are as follows: (1) peer-reviewed original studies evaluating the efficacy, safety, ethical compliance, or cost-effectiveness of MDA, tMDA, and fMDA for malaria control; (2) systematic reviews and Meta-analyses related to the antimalarial strategies of MDA; and (3) operational reports issued by the World Health Organization that provide key data on MDA implementation in tropical regions. The exclusion criteria are: (1) literature with incomplete data or unvalidated research methods; (2) conference abstracts, editorials, and commentaries without original data; (3) duplicate publications; and (4) literature with unavailable full text.

The literature screening process was performed by two independent researchers, who first conducted an initial screening of titles and abstracts, followed by a full-text assessment of potentially eligible studies. In addition, the researchers manually searched the reference lists of included studies and relevant reviews to identify additional relevant research.

## 3. Core Research Dimensions of MDA Anti-Malaria Strategy

### 3.1. Historical Context and Theoretical Foundations of MDA

The WHO defines MDA as the provision of a complete antimalarial treatment course to the entire or most of a population in a defined geographic area in a synchronized manner, without relying on individual test results [2]. Compared with other strategies, MDA offers irreplaceable additional value and a clear complementary mechanism, which can effectively make up for the critical shortcomings of single intervention measures [16,17]. LLINs and IRS can block the mosquito-borne human transmission route, whereas MDA directly targets asexual and gametocyte malaria parasites in the entire human population [8]. In a cluster-randomized trial, the combination of MDA and LLINs reduced malaria incidence by more than 75%. Furthermore, the long-term implementation of MDA combined with other strategies in Comoros and São Tomé and Príncipe cut malaria incidence to zero deaths. In addition, current malaria vaccines show unsatisfactory efficacy and are only suitable for children; they induce protective immunity slowly and have almost no effect on asymptomatic infections. However, MDA can rapidly and prophylactically eliminate malaria parasites before the vaccine immunity takes effect, achieving full population coverage and thus providing crucial complementary value. The integrated strategy of MDA plus vaccines can achieve the dual effects of rapid parasite clearance and the establishment of long-term immune barriers. MDA is also an intervention capable of systematically eliminating asymptomatic carriers, who are the source of residual malaria transmission. It can quickly contain outbreaks in emergency scenarios, a capability that slow-acting vaccines and lagging vector control measures cannot provide. Simultaneously, artemisinin-based treatment regimens have excellent thermal stability, enabling MDA to be implemented in tropical regions with weak health systems and no cold chain infrastructure, breaking through the distribution barriers faced by vaccines and complex vector control tools. In the 1950s, the WHO’s Global Malaria Eradication Program (GMEP) primarily relied on DDT indoor residual spraying (IRS) [18]. Various countries and research institutions attempted to use MDA as a rapid-acting eradication tool. But early attempts, such as the Garki project in Nigeria [19], demonstrated that MDA alone was ineffective under conditions of high basic reproduction numbers and insufficient coverage or persistence. Moreover, decades of subsequent practice have repeatedly confirmed that MDA is highly prone to transmission resurgence in the absence of a supporting prevention and control system, and the sustainability of single interventions is extremely poor, further solidifying the WHO’s cautious and even negative stance toward MDA over the following decades.

### 3.2. Regional Variations in MDA Implementation in Tropical Regions

The implementation of MDA in tropical regions is not static but is profoundly influenced by local epidemiology, socio-cultural factors, and health systems, exhibiting significant regional heterogeneity [20] (Table 1). In high-transmission areas, predominantly in sub-Saharan Africa, dual challenges of resource constraints and adherence issues prevail, with MDA often employed as an emergency measure to suppress outbreaks. However, the implementation failure rate remains persistently high, which has become the core shortcoming of applying this strategy in high-transmission areas. In low-transmission areas of the Greater Mekong Subregion (GMS), challenges of drug resistance and mobile populations emerge [21]. The GMS region has entered the elimination phase, where MDA primarily manifests as targeted mass drug administration (tMDA) for high-risk populations and focal mass drug administration (fMDA) targeting focal points. The primary challenge lies in the high prevalence of artemisinin and combination drug resistance, which limits drug options. Another major obstacle is the cross-border mobile population, characterized by high mobility, poor traceability, and extremely low medication adherence, creating elimination blind spots [22]. Additionally, in tropical regions of South America [23], severe ecological and cross-border transmission challenges, along with unique environmental conditions that complicate Anopheles mosquito control, often constrain MDA implementation due to complex cross-border population movements and low political commitment. In some areas, the lack of sustained financial support leads to intermittent MDA programs, making it difficult to consolidate achievements. While there are successful experiences in Tanzania, Zambia, China, and other places [24], the failure of the above-mentioned tropical indigenous cases and the controversial evidence suggest that MDA may struggle to achieve its intended outcomes if it ignores the influence of factors such as socio-cultural background and health system capacity.

### 3.3. Key Implementation Factors and Cost-Effectiveness

The implementation of MDA has achieved remarkable results in significantly reducing the incidence of malaria, and its success relies on standardized procedures, community participation, and precise cost control [25]. Firstly, community mobilization is the cornerstone. Given the multilingual and multicultural characteristics of tropical regions, it is essential to conduct culturally adapted health education through local health workers and establish a transparent feedback mechanism [26]. It is necessary to popularize the purpose of MDA, the function of drugs, and precautions among the population, and establish a community feedback mechanism to enhance trust. The issue of cost-effectiveness is one of the core controversies regarding the value of MDA [27]. In high-transmission areas, the short-term epidemic suppression value of MDA may offset its costs. For example, in São Tomé and Príncipe, an African country that implemented the MDA strategy, the median direct economic burden of outpatient services decreased from approximately $8.74 to $5.33, the median indirect economic burden decreased from approximately $54.97 to $32.34, the median overall economic burden decreased from approximately $76.75 to $38.80, and the total number of workdays lost by patients and their relatives decreased by 5 days [28]. In addition, in the Comoros, another African country that implemented the MDA strategy, the national direct economic burden per case decreased from $17.97 to $17.68, the indirect economic burden (estimated based on household monthly income) decreased from $7.25 to $6.15, the total economic burden decreased from $24.16 to $22.25, the national overall economic burden of malaria decreased from $475,517.12 to $101,148.50 (a decrease of 78.73%), and the overall economic burden of malaria on Grande Comore decreased from $469,984.48 to $99,568.75 (a decrease of 78.81%) [29]. In low-transmission areas, although tMDA and fMDA are theoretically more cost-effective due to their clear targets, they increase the costs of screening and targeting. According to relevant research data (Table 2), in tropical low-income countries, the per capita cost of full-population MDA per treatment is approximately $4–8, while the per capita cost of tMDA, including screening, may rise to more than $15 [30], which poses a huge challenge to the health budgets of resource-poor countries. The compound artemisinin developed by China (often referred to as “Yue Te Kuai”) provides an example for solving the problem of drug distribution in tropical regions, and is often integrated with projects such as SMC to reduce costs and improve efficiency [31]. Addressing the widespread high-temperature environment of 35–45 °C and the lack of cold chain in Africa, “Yue Te Kuai” has strong thermal stability. Accelerated stability tests show that when stored for 6 months under conditions of 40 °C ± 2 °C and relative humidity of 75% ± 5%, the content of its main components, dihydroartemisinin and piperaquine, decreases by less than 3%, the increase in related substances meets international pharmacopeia standards, and there are no obvious changes in physical properties. In the short-term exposure test to extremely high temperatures of 50 °C (for 7 days) simulating extreme transportation conditions, its active ingredients remain stable with no significant degradation [32]. The specific process of its joint implementation with the African SMC project is as follows: after unified planning at the national level, “Yue Te Kuai” compound artemisinin is used as the MDA drug for people over 5 years old, and is distributed to grassroots levels before the rainy season using the existing logistics system of the SMC project. During the implementation of SMC, CHWs who have received dual training, while visiting households to administer SMC drugs to children under 5 years old, simultaneously distribute and guide the full-course administration of “Yue Te Kuai” to members over 5 years old in the family, achieving an integrated intervention of “one household visit, full family coverage”, and unified monitoring and recording are carried out [33]. In practices in countries such as Nigeria, the “Yue Tekuai” MDA project is often integrated with the existing logistics channels of SMC, using SMC’s distribution network to administer drugs to the entire population before the peak of the rainy season, sharing community distributors, which significantly reduces implementation costs and improves coverage. The coverage rate has reached 85% and above in countries such as Mali, Guinea, and the Comoros.

### 3.4. The Balance Between Security and Ethical Management

Safety and ethics are the core cornerstones for the sustainable advancement of MDA [43,44]. The key lies in constructing a full-process guarantee system through precise risk prevention and control, as well as inclusive ethical practices. Firstly, chloroquine, as a core drug in early MDA, has safety controversies mainly focused on the cumulative toxicity of long-term use: the safety risks of sulfadoxine–pyrimethamine combination preparations are mainly allergic reactions and blood system abnormalities; the core safety hazard of primaquine is hemolytic reactions; ACTs have overall good tolerance, with common adverse reactions being nausea and diarrhea; and the main adverse reactions of ivermectin are neurological reactions and gastrointestinal discomfort. In addition, during the anti-malarial process of artemisinin-based drugs [45], the team has built a full-cycle protection system based on the pharmacological properties of artemisinin-based drugs, the distribution of Glucose-6-phosphate dehydrogenase (G6PD) genotypes, and population baseline health data such as the spectrum of underlying diseases. Furthermore, MDA mostly targets blood-stage parasites and fails to eradicate Pv liver-stage hypnozoites, but the addition of 8-aminoquinoline drugs conflicts with the risk of hemolysis in G6PD deficiency, making MDA in Pv situations more complex [46,47]. Thus, individualized adaptations are implemented for special groups such as children, pregnant women, and the elderly. For children, easy-to-take formulations such as artemisinin-based dispersible tablets and suspensions are used, with precise dosage based on body weight. Pregnant women should receive priority use of pregnancy category B artemisinin combination preparations. When primaquine is used in combination, hemolysis risks are avoided through G6PD-stratified screening or low-dose initiation mode [48]. Secondly, the ethical controversies of MDA focus on the conflict between individual autonomy and public health interests, especially the ethical considerations of requiring non-infected individuals to take drugs in tropical low-transmission areas [49,50]. This controversy is not merely a matter of superficial informed consent but involves the alienation of individuals’ right to health caused by public health interventions, and its ethical legitimacy in low-prevalence areas has not yet been universally recognized by the academic community. This study summarizes the ethically acceptable practical requirements for MDA as follows: (1) base it on solid evidence of public health necessity; (2) ensure that the community is fully informed and that collective or individual informed consent is obtained as much as possible; (3) establish an adverse reaction monitoring and compensation mechanism; and (4) guarantee the right to withdraw. Successful MDA projects all place community participation at the core, transforming it from an intervention implemented on the community to an intervention implemented together with the community.

### 3.5. Regional Analysis of Drug Resistance Risks in Tropical Regions

The relationship between MDA and drug resistance is complex, and irregular drug use is the main driving factor [51,52]. In tropical regions, the risk of drug resistance exhibits significant regional disparities, necessitating precise interventions (Table 3) [53,54,55]. The GMS serves as the epicenter of multidrug resistance worldwide [56,57]. The mutation rate of the Plasmodium falciparum Kelch13 (PfK13) gene exceeds 50% in certain hotspot areas, often coexisting with pyromethamine resistance markers [58]. Implementing MDA in this region must rely on the latest molecular surveillance data, avoiding the use of ACT combinations that have developed severe resistance, and instead opting for alternatives such as mefloquine-artesunate or pyronaridine-artemisinin. Although sub-Saharan Africa as a whole maintains relatively good sensitivity to ACTs, PfK13 mutations (e.g., R561H, P574L) have been independently detected in recent years in Rwanda, Uganda, and other regions, with mutation rates in some areas approaching the 5–10% warning threshold [59]. Additionally, drug resistance has emerged in other regions, and there is considerable debate among scholars regarding the mechanisms of resistance development [60,61]. Although widespread clinical treatment failure has not yet occurred, large-scale MDA may exert selective pressure. Therefore, strict molecular marker surveillance must be conducted before and after MDA implementation, in accordance with the WHO malaria guidelines.

### 3.6. Limitations and Countermeasures for the Implementation of MDA in Tropical Regions

Despite the considerable potential of MDA to rapidly reduce disease burden, its practical application in tropical regions remains constrained by several limitations (Table 4) [67,68,69,70,71]. (1) High resource dependence and limited long-term sustainability: In high-prevalence areas, discontinuation of MDA may be linked to a relatively rapid rebound in disease outbreaks, especially where strong vector control systems and reliable health system support are lacking. (2) Community fatigue and declining treatment compliance: In regions requiring multiple rounds of medication, as perceived local disease risk diminishes, medication adherence tends to drop noticeably, with this pattern particularly evident among adult males. (3) Asymptomatic infections and under-detection: Even following high-coverage MDA, individuals with low-density asymptomatic parasitemia may go undetected, potentially acting as residual sources for renewed malaria transmission. (4) Long-term ethical and resistance risks: Ethical controversies surrounding MDA use in healthy populations and the potential risk of accelerating drug resistance persist. The targeted solution can be summarized as implementing an “MDA + seasonal prophylaxis” combination in high-transmission areas to rapidly reduce malaria incidence and improve cost-effectiveness and efficiency. Additionally, in low-transmission areas, “precision screening + targeted intervention” is adopted to avoid community resistance and enhance prevention and control efficacy.

### 3.7. Synergistic Effects with Integrated Prevention and Control Systems

A variety of evidence-based interventions have been validated to reduce malaria incidence across different transmission settings [72]. LLINs and IRS, as core vector control tools, reduce malaria incidence by 40–50% and 30–40%, respectively, by blocking mosquito-to-human transmission. Malaria vaccines RTS, S/AS01, and R21/Matrix-M achieve a 75% incidence reduction when deployed seasonally and 39–67% under routine administration but are limited to children under 5 years old and require 4–6 weeks to induce protective immunity. SMC reduces clinical malaria incidence by 50–71% in highly seasonal transmission regions but only targets young children. In contrast to other interventions, MDA demonstrates unique and context-adaptable efficacy in reducing malaria incidence and interrupting transmission. MDA has low costs and minimal coverage difficulties and also imposes low requirements on health workers. Currently, the efficacy of malaria vaccines is only 30–40%; vaccines are expensive, have high requirements for health workers during administration, and are difficult to store. In several empirical studies, we have also found that vaccines are relatively beneficial only among children, whereas MDA can substantially reduce the prevalence across entire communities, especially among high-risk individuals aged 18–45. MDA requires deep integration and synergistic efforts with the IPC system. Combined with vector control LLINs and IRS, which provide sustained transmission barriers, this approach can significantly prolong protective effects and prevent reinfection after MDA [73]. When synergized with highly sensitive diagnostic technologies, such as ultra-sensitive RDT or nucleic acid testing, it can compensate for the shortcomings of conventional RDT in detecting low-density infections, thereby enhancing the accuracy of tMDA [74,75]. Collaborative efforts with cross-border governance, including synchronized MDA in border areas, coupled with information sharing and joint vector control, are crucial for interrupting imported transmission and consolidating elimination achievements [76].

### 3.8. Future Strategy of MDA+

Malaria remains one of the most significant global public health challenges [77], with alarmingly high prevalence in several African countries. The resurgence of malaria in nations like the Comoros has created substantial pressure [78]. The future of MDA lies in evolving into a precision-driven, dynamic “MDA+” integrated strategy. “MDA+” adheres to the core principles of data-driven decision-making, multi-tool collaboration, full-process closed-loop management, and long-term sustainability [79]. It dynamically adjusts medication regimens based on drug resistance surveillance, integrates innovative vector control, single-dose radical cure drugs, and malaria vaccines to establish a dual defense line of immune protection and parasite elimination. Combined with precision diagnosis and treatment, it enhances drug resistance early warning, focuses on community participation, financing mechanisms, and health system capacity, forming a complete operational system [80]. The implementation framework consists of four steps: (1) precise analysis and customized planning, including stratification and zoning based on baseline surveys and drug resistance surveillance, optimized drug selection, and completion of health education and ethical safeguards; (2) stratified and precise drug administration, with MDA+SMC+LLINs used in high-transmission areas, tMDA/fMDA combined with active case detection in low-transmission areas, and individualized dosing for special populations; (3) full-process dynamic monitoring, using digital platforms to track prevention and control indicators, monitor drug resistance genes, and manage medication adherence; and (4) consolidation of elimination and long-term prevention and control, combining vaccines, vector control, and cross-border collaboration to block imported transmission and sustain malaria elimination achievements.

MDA+ has been implemented and verified in multiple countries [81,82,83,84,85]: Comoros has adopted heat-resistant artemisinin combined with vector control, achieving a more than 90% reduction in malaria cases in high-transmission areas and interrupting local transmission; Nigeria has carried out MDA by integrating SMC networks, attaining low-cost and high coverage with a 75% drop in incidence; Cambodia has implemented fMDA relying on digital surveillance and drug resistance management, successfully blocking falciparum malaria transmission in low-transmission areas; and Tanzania has rapidly suppressed the epidemic through MDA combined with LLINs, leading to a 75% decrease in case incidence. MDA+ can strengthen drug resistance early warning in coordination with precise diagnosis and treatment, and further research is needed on deepening community participation, financing, and adaptation of health systems. This strategy is still in the exploratory stage, and more empirical studies are required to improve its synergistic combinations, long-term prevention and control effects, and large-scale sustainability.

## 4. Summary

Despite challenges such as localized situation reversals, drug resistance, and implementation difficulties, MDA remains a critical option in the malaria elimination toolkit for tropical regions. The rational application of MDA depends on transmission intensity, health system capacity, resource availability, and community acceptance. The key to the future lies in acknowledging its limitations and adopting tailored “MDA+” precision and integration strategies, MDA+ vaccine, upgraded MDA+ vector control, and MDA+ digital compliance tools to enhance medication supervision efficiency and improve overall prevention and control resilience, particularly in high-prevalence areas like Africa, where more attention should be paid to implementation research based on local socio-cultural contexts to optimize interventions and delay drug resistance emergence. Furthermore, the development of drug resistance is multifaceted, necessitating a combination of multiple strategies to prevent or delay its occurrence. We must proactively address future malaria control approaches, enhance targeted research on antimalarial drugs, improve the efficacy of malaria vaccines, and strengthen efforts to eliminate the impact of all malaria species and the recently described Plasmodium knowlesi, with a view to achieving the goal of complete malaria eradication [86,87].

## Figures and Tables

**Table 1:** The regional disparities in MDA implementation in tropical regions.

Region	Sub-Saharan Africa (High Transmission, Resource-Poor)	Greater Mekong Subregion (Low Transmission, High Prevalence of Drug Resistance)	South America (Transboundary Spread Prominent)
MDA application condition	It is intended for use in the emergency suppression of outbreaks or rapid reduction in community parasite loads prior to seasonal transmission peaks, as a complement to vector control.	MDA is no longer recommended for the general population. It is primarily indicated for targeted clearance in specific high-risk populations (tMDA) or localized outbreak foci (fMDA).	It is suitable for local intervention in hard-to-reach cross-border migrant populations, specific occupational groups such as mining/forestry areas, or border transmission hotspots.
Core barriers and challenges	Weak healthcare system: Disrupted supply chains, insufficient staffing, and chaotic data management. Community acceptance: Differences in cultural beliefs, concerns about drug side effects, and poor adherence to multiple rounds of medication. Resource limitations: Significant gaps in drug and operational costs.	Multidrug resistance: Particularly resistance to ACTs combination drugs (e.g., mebendazole and methoxyflurane) limits drug selection. Migrant population management: High mobility of cross-border workers and forest workers makes it extremely difficult to track and complete treatment courses. Residual transmission: Relapses caused by _Pv_ dormant subpopulations are difficult to resolve through conventional MDA.	Geography and Ecology: The vast Amazon rainforest features complex terrain and poor transportation access, with unique mosquito ecosystems that are difficult to control. Cross-border Coordination: Uneven political commitments, prevention strategies, and resource allocation among countries hinder the establishment and maintenance of cross-border joint prevention and control mechanisms. Social Instability and Illegal Activities: Illegal mining and other activities in certain regions increase the difficulty of intervention and safety risks.
Localization solutions	Integration strategy: Combine MDA with seasonal malaria chemoprevention (SMC) or vaccination programs, sharing distribution networks. Community empowerment: Utilize community health workers (CHWs) and traditional leaders for social mobilization and drug distribution. Innovative tools: Adopt heat-resistant drug formulations (e.g., “Yue Te Kuai”) for high-temperature environments.	Precision Guidance: Utilize molecular surveillance and GIS to identify hotspots and implement tMDA and fMDA strategies. Adjustment of regimens: Reconfigure antimalarial combination therapies (e.g., pyronaridine-artemisinin) based on drug resistance data, and explore the introduction of single-dose radical cure agents (e.g., thienofluoroquine) for Pv. Cross-border collaboration: Establish regional information-sharing platforms and joint action mechanisms, and set up border medical stations for mobile populations.	Multi-sector collaboration: Joint military and non-governmental organization (NGO) efforts to implement interventions in remote areas. Regional initiatives: Leveraging frameworks such as the Pan American Health Organization (PAHO) to advance cross-border malaria elimination programs. Focus on mobility: Developing innovative service models for mobile populations, including mobile clinics, peer educator networks, and integrating with long-term vector control tools.

**Table 2:** A comparison of the cost parameters for MDA, tMDA, and fMDA in different tropical regions.

Strategy	Applicable Context	Estimated Cost per Treatment Completed, USD	Key Cost Drivers	Benefit Characteristics
MDA	High transmission areas, emergency containment of outbreaks	$4.00–$8.00	Drug procurement, large-scale logistics, and the mobilization of all staff for distribution	The incidence and mortality rates can be rapidly reduced in a short period of time, with significant scale effects.
tMDA	Low transmission areas/elimination phase, targeting high-risk subgroups	$12.00–$20.00+	Screening and location costs (requiring initial identification of target populations) and labor costs for tracking migrant populations	Reducing drug waste and targeting infection sources precisely, but with high upfront identification costs.
fMDA	Low transmission areas, targeting identified outbreak villages or regions	$8.00–$15.00	Hotspot identification and monitoring system and regional strengthened mobilization	Local areas should be cleared quickly to prevent the spread of the epidemic.
MDA + SMC Integration Mode	High seasonality transmission area, targeting the entire population (using SMC networks)	$3.00–$6.00 (Lower marginal cost)	Hotspot identification and monitoring system, regional strengthened mobilization	The high cost-effectiveness ratio enables synergy through existing platforms.

Note: The comprehensive estimation range is based on the literature [34,35,36,37,38,39,40,41,42].

**Table 3:** The distribution differences in drug resistance genes across regions [62,63,64,65,66].

Region	Type	PfK13 Key Validation Mutation Prevalence	Status of Partner Drugs Used in Combination with ACT
Core resistance area	GMS (e.g., Cambodia, Thailand, Myanmar, border areas of Vietnam)	High (>15–50%+) (Common mutations: C580Y, Y493H, R539T, etc.)	Severe multidrug resistance
New threat area	Hotspots in East Africa (e.g., Rwanda, Uganda, parts of Tanzania)	Moderate/ascending (5–15%) (increased specific local mutations, such as R561H, A675V)	Still sensitive, but under threat
Relatively sensitive area	Most of sub-Saharan Africa (West Africa, Central Africa, and most of the high transmission areas in South Africa)	Low (<5%) (rare or below WHO alert in most areas)	Still sensitive
Other areas	The Amazon basin in South America and other regions	Local presence (sporadic >5%) (There is an independent origin of C580Y)	High variability

**Table 4:** The limitations and coping strategies of MDA in tropical regions.

Limitation Category	Specific Manifestation in Tropics	Proposed Coping Strategies
Socio-cultural and Adherence	Tribal cultural barriers, distrust in modern medications, community fatigue caused by multiple rounds of medication, and challenges in reaching migrant populations.	Enhance community participatory design: Mobilize traditional leaders and local opinion leaders, adopt culturally appropriate outreach methods, transition to targeted tMDA in low-communication areas to minimize disruption to the general population.
Health System and Resources	The high dependence on resources and the extreme difficulty of logistics in remote tropical areas, as well as the frequent interruption of projects due to the discontinuation of external funding, have led to a resurgence of the epidemic.	Integration and Innovation: Utilize heat-resistant drugs to overcome cold chain bottlenecks, integrate MDA with existing SMC and EPI projects to share costs, and secure domestic fiscal commitments to reduce dependence on external single funding sources.
Biological and Technical	The inability to eradicate low-density infections of the asexual phase of Pf and dormant Pv parasites, the sensitivity of rapid diagnostic tests (RDT) is insufficient to support precise localization, and the potential risk of drug resistance.	Combination Toolkit: MDA must be combined with enhanced vector control, explore the use of single-dose tafquinine for eradication of Pv, introduce ultra-sensitive detection techniques to guide fMDA, and strictly monitor resistance markers.
Ethical Considerations	The ethical legitimacy of administering drugs to a large number of healthy, non-infected individuals in low-prevalence areas is questioned.	Transparency and precision: Establish robust informed consent mechanisms for communities and individuals, clarify the public health necessity of MDA, and, where possible, prioritize screening-based strategies over population-wide MDA to balance individual risks with collective benefits.

## Data Availability

Summary of the literature data reports.
